# National Association of Dental Faculties

**Published:** 1897-09

**Authors:** 


### NATIONAL ASSOCIATION OF DENTAL FACULTIES.

THE Fourteenth Annual Meeting of the National Association of Dental Faculties was held at the Hygeia Hotel, Old Point Comfort, Va., commencing Friday, July 30, 1897.

The following colleges of the Association were represented as noted below:

*Alabama Dental College*, Birmingham, Ala.—T. M. Allen.

*University of California, Dental Department*, San Francisco, Cal. —L. L. Dunbar.

*Columbian University, Dental Department*, Washington, D. C.— J. Hall Lewis.

*Howard University, Dental Department*, Washington, D. C.—A. J. Brown.

*National University, Dental Department*, Washington, D. C.—J. Roland Walton.

*Atlanta Dental College*, Atlanta, Ga.—William Crenshaw.

*Dental Department of Southern Medical College*, Atlanta, Ga.— S. W. Foster.

*Chicago College of Dental Surgery*, Chicago, Ill.—T. W. Brophy, Louis Ottofy.

*Northwestern University Dental School*, Chicago, Ill.—Theo. Menges.

*State University of Iowa, Dental Department*, Iowa City, Iowa.— W. S. Hosford.

*Louisville College of Dentistry*, Louisville, Ky.—H. B. Tileston.

*Baltimore College of Dental Surgery*, Baltimore, Md.—M. W. Foster.

*University of Maryland, Dental Department*, Baltimore, Md.—F. J. S. Gorgas.

*Boston Dental College*, Boston, Mass.—J. A. Follett.

*Harvard University, Dental Department.*—Thos. Fillebrown.

*Dental College of the University of Michigan*, Ann Arbor, Mich.—J. Taft.

*University of Minnesota, Dental Department*, Minneapolis, Minn.—W. P. Dickinson.

*Kansas City Dental College*, Kansas City, Mo.—J. D. Patterson.

*Western Dental College*, Kansas City, Mo.—D. J. McMillen.

*Marion-Sims College of Medicine, Dental Department*, St. Louis, Mo.—J. H. Kennerly.

*Missouri Dental College*, St. Louis, Mo.—A. H. Fuller.

*University of Buffalo, Dental Department*, Buffalo, N. Y.—W. C. Barrett.

*New York College of Dentistry*, New York City.—F. D. Weisse, J. Bond Littig.

*Cincinnati College of Dental Surgery*, Cincinnati, Ohio.—G. S. Junkermann.

*Ohio College of Dental Surgery*, Cincinnati, Ohio.—H. A. Smith.

*Western Reserve University, Dental Department*, Cleveland, Ohio.—George H. Wilson.

*Pennsylvania College of Dental Surgery*, Philadelphia, Pa.—C. N. Peirce.

*Philadelphia Dental College*, Philadelphia, Pa.—S. H. Guilford, Leo Greenbaum.

*University of Pennsylvania, Dental Department*, Philadelphia, Pa.—James Truman.

*Tennessee Medical College, Dental Department*, Knoxville, Tenn.—R. N. Kesterson.

*Central Tennessee College, Meharry Medical Department, School of Dentistry*, Nashville, Tenn.—G. W. Hubbard.

*University of Tennessee, Dental Department*, Nashville, Tenn.—J. P. Gray, L. G. Noel.

*Vanderbilt University, Dental Department*, Nashville, Tenn.—H. W. Morgan.

*University College of Medicine, Dental Department*, Richmond, Va.—L. M. Cowardin.

*Royal College of Dental Surgeons*, Toronto, Canada.—W. E. Willmott.

The following colleges were elected to membership:

*Milwaukee Medical College, Dental Department*, Milwaukee, Wis., represented by Reinhold E. Maercklein.

*Tacoma Dental College*, Tacoma, Wash., the constitution being signed by proxy by Dr. Kennerly.

*New York Dental School*, New York City, represented by John I. Hart.

*Ohio Medical University, Dental Department*, Columbus, Ohio, represented by J. F. Baldwin.

*Baltimore Medical College, Dental Department*, Baltimore, Md., represented by J. W. Smith and William A. Montell.

The application for membership of the University of Omaha, Dental Department, was laid over till next year, at the request of its officers.

Applications for membership were reported by the Executive Committee from the Pittsburg Dental College, Pittsburg, Pa.; Dental Department of the College of Physicians and Surgeons, San Francisco, Cal.; Colorado School of Dentistry, Denver, Col.

The following report laid over from last year was adopted:

> "Your committee on choosing a color respectfully report that they have decided to recommend the standard lilac as the distinctive dental color, and they recommend the adoption of the academic costume according to the requirements observed by the intercollegiate system."

The resolutions laid over from last year, making the annual college term seven full months, and recommending that the annual meetings be held in connection with the National School of Dental Technics, and at a time of the year when the colleges are in session, were negatived.

A committee, consisting of Drs. Henry W. Morgan, M. W. Foster, Theo. Menges, C. N. Peirce, and H. A. Smith, was appointed to meet a similar committee from the National Association of Dental Examiners, for the purpose of harmonizing the differences of opinion between the two associations. This committed reported rules which had been agreed upon by the two committees.

The report was discussed at length and again referred to the committee, which later reported, through the Executive Committee, a resolution, which was adopted, providing for the codifying and arranging of the existing rules of the Association, and the preparation of such additional rules as may be deemed advantageous to both organizations in advancing the standard of dental education in the United States. On motion, the committee which had had the matter in charge in the conference was continued for this purpose.

A communication from the Dental Department of the State University of Iowa was received, asking consent of the Association to its conferring the honorary degree on Dr. F. P. Weber, of Cherokee, Iowa. The request was declined on the ground that it is contrary to the practice of the Association.

A similar communication from the University College of Medicine, Dental Department, Richmond, Va., asking the privilege of conferring the *ad eundum* degree on Dr. Thomas G. Cowardin, of London, Eng., was refused upon the same grounds.

The rule regarding preliminary qualifications adopted in 1896 was declared to have been adopted in an unconstitutional manner, and was therefore rescinded. The following was adopted in its place, and by unanimous consent was ordered to go into effect at once:

*Resolved*, That the minimum preliminary education requirement of a college of this Association shall be a certificate of entrance to the first year of a high school or—in States that have no high school—of graduation from a grammar school, or its equivalent, to be determined by an examination.

*Resolved*, That nothing in the above shall be construed to interfere with colleges of this Association that are able to maintain a higher standard of preliminary education.

A communication was read from Dr. W. Mitchell, president of the American Dental Club of London, requesting the appointment of a committee to co-operate with a similar committee in Europe for the purpose of securing just recognition of the diplomas issued by the colleges belonging to the Association. The communication was favorably considered, and the president appointed as the committee, Drs. W. C. Barrett, D. J. McMillen, S. H. Guilford, A. H. Fuller, and Faneuil D. Weisse.

The *Ad Interim* Committee reported that one new question decided by them during the year was that a student who was in arrears for fees could not be accepted by another college if objection was made by the college to which he was indebted. This ruling was sustained by vote of the Association.

The committee also recommended that steps be taken to secure definite knowledge as to the curricula and requirements of foreign colleges, so that the members of the Association should be able to decide upon the standing of students coming from them. Referred to the committee appointed to consider the matter of Dr. Mitchell's letter.

A paper prepared by Dr. W. C. Barrett, Buffalo, N. Y., at the request of the Executive Committee, and entitled "The Study of Anatomy," was read by its author.

(For Dr. Barrett's paper, see page 581.)

The paper was, on motion, directed to be incorporated in the official report and copies sent to the journals for publication.

A committee, consisting of Drs. S. H. Guilford, Theo. Menges, and M. W. Foster, was appointed to select persons to prepare papers on subjects connected with the work of the Association, to be read before the next meeting.

Dr. Barrett offered the following, which was adopted:

*Resolved*, That the final vote upon the admission of a college to this Association shall not hereafter be taken unless a duly certified and qualified delegate is in attendance.

The following resolution, offered by Dr. L. L. Dunbar, was adopted:

*Resolved*, That in order to maintain a reputable standing in this Association, no college under its jurisdiction shall permit any member of its faculty or teaching staff, board of trustees, or stockholders to serve in a judicial capacity as a member of a State Board of Examiners.

Dr. Taft offered the following, which was adopted:

*Resolved*, That a committee of three on curriculum be appointed, whose duty it shall be to compare the schemes of study of the various dental colleges, with the view of harmonizing these schemes and making them as nearly alike as practicable, to report next year.

The Committee on Text-Books recommended the following:

Essig's "American Text-Book of Prosthetic Dentistry."

Hodgen's "Dental Metallurgy."

Schafer's "Essentials of Histology," fourth edition.

Abbott's "Principles of Bacteriology," third edition.

Gray's "Anatomy," last edition.

Luff's "Manual of Chemistry."

Burchard's "Compend of Dental Pathology and Therapeutics."

The report was adopted, and the committee was instructed to examine Kirk's "American Text-Book of Operative Dentistry,"

and Marshall's "Injuries and Surgical Diseases of the Face, Mouth, and Jaws," and forward their views at the earliest possible moment to the secretary, in order that they may be incorporated in the printed Transactions.

A committee, consisting of Drs. M. W. Foster, William Crenshaw, and L. G. Noel, reported appreciative resolutions on the death of Drs. Frank Abbott and Francis Peabody, late members, who have died since the last meeting was held. The resolutions were adopted.

The following lie over for final action till next year:

Offered by Dr. H. W. Morgan, seconded by Dr. H. B. Tileston:

*Resolved*, That on and after the session of 1899–1900, the regular sessions of each college belonging to this Association shall be extended to four years.

Dr. J. Taft moved to amend the constitution to require applications for membership to be sent to the secretary of the Executive Committee instead of to the secretary of the Association.

Offered by Dr. T. Fillebrown:

*Resolved*, That no college connected with this Association shall confer any degree as honorary which is usually granted in due course of study and examination. All former rules on the subject are hereby repealed.

Offered by Dr. Barrett:

*Resolved*, That after the regular session of 1898–99 the annual college term for the members of the Association shall be seven full months.

Dr. Crenshaw moved to strike out Rule 3 and adopt the following instead:

*Resolved*, That the time in which students can enter schools of this Association shall be the first ten days of the session of the school, dating from the time announced in its catalogue.

The following were elected officers for the ensuing year: T. W. Brophy, Chicago, President; D. J. McMillen, Kansas City, Mo., Vice-President; J. H. Kennerly, St. Louis, Mo., Secretary; H. W. Morgan, Nashville, Tenn., Treasurer.

*Executive Committee.*—J. Taft, Cincinnati; Thomas Fillebrown, Boston, Mass.; B. Holly Smith, Baltimore, Md.

*Ad Interim Committee.*—James Truman, Philadelphia; F. J. S. Gorgas, Baltimore; J. Hall Lewis, Washington, D. C.

The newly elected president, on being installed, announced the following appointments:

*Committee on Schools.*—J. A. Follett, Boston, Mass.; H. A. Smith, Cincinnati, Ohio; L. L. Dunbar, San Francisco, Cal.; J. D. Patterson, Kansas City, Mo.; W. T. McLean, Cincinnati, Ohio.

*Committee on Text-Books.*—S. H. Guilford, Philadelphia, Pa.; William Crenshaw, Atlanta, Ga.; W. C. Barrett, Buffalo, N. Y.; W. P. Dickinson, Minneapolis, Minn.; Faneuil D. Weisse, New York City.

*Committee to select Subjects and Essayists for Next Meeting.*—J. Taft, Cincinnati, Ohio; Edward C. Kirk, Philadelphia, Pa.; A. H. Fuller, St. Louis, Mo.

Adjourned to meet at the call of the Executive Committee.

